# Uncemented hip resurfacing in patients over 65: 16-year outcomes from a large, single-surgeon series

**DOI:** 10.1007/s12306-025-00929-2

**Published:** 2025-11-06

**Authors:** D. Gaillard-Campbell, T. Gross

**Affiliations:** Midlands Orthopaedics & Neurosurgery, PA, Columbia, SC USA

**Keywords:** Hip resurfacing arthroplasty, Older adults, Metal-on-metal, Long-term survivorship, Femoral neck fracture

## Abstract

**Background:**

Hip resurfacing is often reserved for younger, active patients due to concerns over implant durability and fracture risk in older patients. However, there is a lack of long-term data evaluating outcomes in patients over 65 years old at time of surgery. This study addresses this gap by directly comparing implant survivorship, failure and complication rates, and clinical outcomes in a younger control cohort from the same large single-surgeon series (> 6000 cases).

**Methods:**

We queried a large, prospective institutional clinical database comprising over 7000 hip resurfacing cases performed by a single, high-volume surgeon; findings may not be generalizable to lower-volume or less-experienced centers. From this cohort, we identified 395 cases in patients ≥ 65 years (mean age 68.8) and 5,106 cases in patients < 65 years of age at time of surgery. All included patients had a minimum of 2-year follow-up, and 88.4% of the study cohort had up-to-date follow-up.

**Results:**

There was no difference in the 98.6% 10- and 98.2% 16-year Kaplan–Meier implant survivorship of our over-65 study group when compared to a younger under-65 control group. These results exceed the NICE (National Institute for Clinical Excellence) criteria and registry benchmarks for total hip replacement from three major registries. The early femoral failure rate (femoral failure occurring < 1 year postoperative) was only 0.3%, which compares favorably with total hip arthroplasty perioperative fracture data. The long-term femoral fracture rate over the duration of this study was 1.3% for this older cohort, lower than what was reported for total hip arthroplasty.

**Conclusions:**

This is the first known long-term study to demonstrate that hip resurfacing patients over 65 can achieve implant survivorship rates equivalent to younger resurfacing patients and superior to national total hip arthroplasty registry benchmarks. These findings challenge age-based restrictions and support expanded candidacy for hip resurfacing in select older adults when managed by experienced surgeons.

**Supplementary Information:**

The online version contains supplementary material available at 10.1007/s12306-025-00929-2.

## Background

Hip resurfacing arthroplasty (HRA) offers a functional advantage when compared to standard stemmed total hip arthroplasty (THA). The advantages derive from two factors: lack of a thigh-pain inducing stem and the presence of a more mechanically stable bearing. This gives HRA patients a more normal feeling hip [1] and a more nearly-normal gait [2–5] compared to THA. Further, HRA allows patients to participate in more vigorous sports (particularly impact sports) and heavy labor [6–9] as well as having less limitations with high range-of-motion (ROM) activities such as ballet, gymnastics, yoga, and kayaking due to a lower risk of dislocation [10]. It has now been amply demonstrated that HRA in the hands of expert surgeons carries a similar and perhaps even lower long-term failure rate in younger patients [11–21]. In the British National Joint Registry (NJR) [22, 23] and the Australian registry [23], durability data are mixed. In the NJR, THA outperforms HRA for durability, but the mean surgeon volume for HRA is only 2.6 cases/year; this is far fewer than that of THA and indicates that many HRA datasets are limited by less-experienced resurfacing surgeons [24]. Further, the data for the infamous DePuy ASR [25] were included in the HRA data, but metal-on-metal (MoM) THA was excluded in the THA data. Both of these factors strongly negatively bias the registry data against HRA. The Australian Registry [23] reports that women have a higher failure rate with HRA at all ages, and men have a higher failure rate if they are over 60 years age. Only men under 60 have a lower failure rate with HRA. Surgeon volume data are not available, but likely follow a similar pattern as in Britain.

Despite registry limitations, other metrics further inform the comparison between HRA and THA. All-cause mortality has been extensively studied and appears to strongly favor HRA, which has been shown to carry a 25–600% lower ten-year mortality when compared to THA in several studies that match confounding factors to varying degrees [26–30].

For women and older patients, there seems to be a trade-off between function and mortality versus implant durability. As our results with HRA have improved from 91% 10-year implant survivorship [31] to 98% 17-year implant survivorship, we have become more confident with this operation, and we have expanded our indications to include older patients. In this study, we examine our prospective clinical database to compare the outcomes in older patients to determine if excluding them from resurfacing is still a practical recommendation.

Although most older patients are satisfied with getting out of pain, resuming daily living activities, and returning to mildly active lifestyle (ex. walks, golf, pickleball), there remains a substantial group who are still dissatisfied [32, 33] and would like to resume more vigorous activities than THA allows.

## Materials and methods

We used a prospective institutional clinical SQL database of over 7000 HRA procedures to retrospectively identify our study group of patients at or over 65 years old at time of surgery. This study group comprised 395 performed between March 2007 and October 2022. For the same date range, we defined our under-65 control group as the remaining 5106 cases. In both groups, the uncemented ReCap™–Magnum™ implant system was employed. In the USA, the ReCap™ and Magnum™ implants are FDA approved but using them as an HRA system is considered off-label use. We published comprehensive metallurgy and design details for these implants previously [20]. Table 1 presents demographic information. Average age at time of surgery was 68.8 years (range 65–85) for the study group and 53.0 (range 12–64) for the control group. The study group had a higher percentage of cases with osteoarthritis (91.1 vs. 79.1%, *p* < 0.0001) and a lower percentage of patients with dysplasia and osteonecrosis (*p* = 0.0004 and 0.0005, respectively), which are diagnoses that typically result in worse outcomes. When compared with the younger control group, the study group had a lower BMI (26.3 vs 27.3, *p* < 0.0001), lower bone density (T-score − 0.3 vs 0.0, *p* < 0.0001), and higher mortality rate (7.3 vs 0.6%, *p* < 0.0001).

**Table 1:** Demographics

Variable	< 65 y.o	≥ 65 y.o	*p* value
Date range	03/2007–10/2022		–
# of cases	5106	395	–
# deceased	30 (0.6%)	29 (7.3%)	< 0.0001*
*Demographics*	–		
% Female	1426 (27.9%)	123 (31.1%)	0.171
UTD Follow-Up (#, %)	3970 (77.8%)	349 (88.4%)	< 0.0001*
Age (Years)	53.0 ± 7.9	68.8 ± 3.4	< 0.0001*
BMI	27.3 ± 4.7	26.3 ± 4.3	< 0.0001*
T-score	0.0 ± 1.3	-0.3 ± 1.2	< 0.0001*
*Diagnoses (#, %)*	–		
Osteoarthritis	4040 (79.1%)	360 (91.1%)	< 0.0001*
Dysplasia	648 (12.7%)	26 (6.6%)	0.0004*
Rheumatoid arthritis	10 (0.2%)	0 (0.0%)	0.379
Post-trauma	62 (1.2%)	2 (0.5%)	0.208
Legg–calve–perthes disease	54 (1.1%)	3 (0.8%)	0.575
Slipped capital femoral epiphysis	20 (0.4%)	0 (0.0%)	0.211
Osteonecrosis	243 (4.8%)	4 (1.0%)	0.0005*
+ Concomitant gluteal tear	33 (0.6%)	17 (4.3%)	< 0.0001*
Other	24 (0.5%)	0 (0.0%)	0.171

Bold, asterisk indicates significant difference

The primary surgeon (TPG) performed all HRA operations through the posterior approach as described previously [34]. Since 2009, normalized to standing intraoperative radiographs have been utilized in all cases to confirm that the acetabular component position is within our validated safe zone (relative acetabular inclination limit (RAIL) [35, 36] to prevent adverse wear-related failure (AWRF). Table 2 presents a summary of surgical information.

**Table 2:** Surgical data

Variable	< 65 y.o	≥ 65 y.o	*p* value
Length of incision (in)	4.2 ± 0.5	4.2 ± 0.4	1.000
Operation time (min)	92.8 ± 25.7	90.7 ± 55.0	0.163
Estimated blood loss (mL)	169.6 ± 126.1	154.9 ± 82.0	0.023*
Hospital stay (days)	1.0 ± 1.0	0.9 ± 0.8	0.052
#Transfusion received	2 (0.04%)	0 (0.0%)	0.718
ASA score	1.8 ± 0.6	2.0 ± 0.6	< 0.0001*
Femoral component size (mm)	49.7 ± 3.5	49.6 ± 3.3	0.583

Bold, asterisk indicates significant difference

We instruct patients to progress to weight-bearing as tolerated unless they present with low preoperative bone density. All patients are tested with a DEXA (dual-energy X-ray absorptiometry) scan preoperatively. Patients at high risk for fracture (bone density T-score < − 1.5) [37, 38] are put on a modified weight-bearing program. Otherwise, patients are instructed to use crutches for two weeks and a cane for two weeks thereafter. We require no formal physical therapy following hospital or ambulatory surgery center discharge. Patients may progress to moderate aerobic exercise at 6 weeks and unlimited activity at six months postoperative. Patients with moderate fracture risk (T-score below zero or BMI > 29) are also placed on alendronate for 6 months.

Since 2007, we have routinely requested metal ion test results at 2 years postoperatively, when run-in wear has been shown to be completed [39]. We also requested metal ion results from all patients with operations prior to 2007 at least once. Overall, 64% of all uncemented resurfacing patients and 93% of the study group followed our recommendation and had ion levels drawn. We used whole blood cobalt (Co) and chromium (Cr) ion values for all comparisons; any serum and plasma laboratory results were converted to whole blood values using Smolder’s method [40]. Based on previous research, we define 5 ion-level categories: normal, optimal [41], acceptable, problematic [42, 43], and potentially toxic [44, 45].

We routinely request that patients return for an office visit or to complete a remote follow-up package at 6 weeks, 1 and 2 years, and every other year thereafter. Each follow-up interval includes a clinical questionnaire, radiographic analysis, and a physical examination testing ROM and strength. Physical examinations are no longer requested of patients after the 1-year postoperative visit for remote follow-ups. We use clinical questionnaires to collect information for calculating the following scores: Harris hip score (HHS) University of California [46], Los Angeles (UCLA) activity score [36], and visual analog scale (VAS) pain scores [37]. We use HHS for quantitative measurement of overall clinical outcome, based on function and ROM. UCLA scores measure patient activity level on a scale of 1 to 10, for which 10 represents regular participation in impact sports. VAS pain scores provide a grade of overall pain on normal and worst days based on a scale of 0 (no pain) to 10 (maximum/debilitating pain). For patients who do not complete the full biennial follow-up questionnaire, we further request a brief response whether their implant is still in place and if they are satisfied, as described by Brooks [16]. This information is presented in Table 3.

**Table 3:** Clinical outcomes

Variable	< 65 y.o	≥ 65 y.o	*p* value
HHS score	97.5 ± 6.6	94.7 ± 10.4	< 0.0001*
Pain	42.4 ± 4.7	42.2 ± 5.3	0.431
Function	85.9	85.7	0.732
UCLA score	7.5 ± 1.9	6.5 ± 2.2	< 0.0001*
VAS Pain: Regular day	0.3 ± 0.9	0.4 ± 1.2	0.04*
VAS Pain: Worst day	1.4 ± 2.1	1.3 ± 2.0	0.373

Bold, asterisk indicates significant difference

**Fig. 1:**
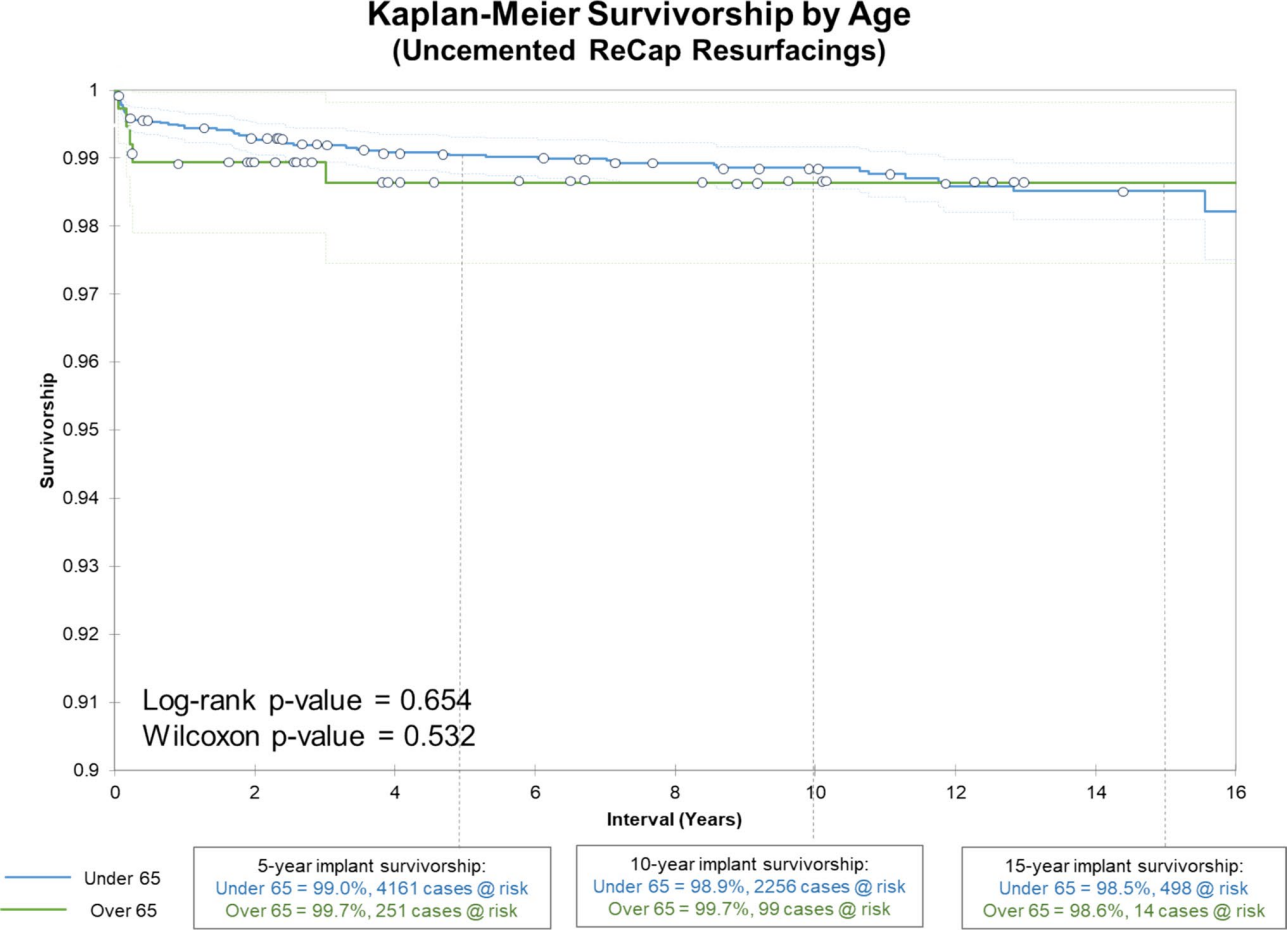
Kaplan–meier implant survivorship

Radiographs are obtained at every follow-up and analyzed for component position, shifting, and radiolucencies. We determine the acetabular inclination angle (AIA) by measuring the angle formed between a horizontal reference line running across the face of the inferior pubic rami and a measurement line running across the face of the acetabular component on the patient’s standing anterior–posterior X-ray (Fig. 1). We grade anteversion using the method of Langton [47]. All measurements were performed using InteleViewer^®^ (InteleRAD, Chicago, IL, USA).

We performed all statistical analyses at a confidence level of 95% using Microsoft^®^ Excel (Microsoft, Redmond, WA, USA) and SAS^®^ (SAS Institute Incorporated, Cary, NC, USA). We utilized paired, 2-tailed Student’s t tests to find significant differences between numeric results, and we compared ratios between groups using a two-sample proportion Z test. Kaplan–Meier (KM) implant survivorship curves were plotted using XLSTAT^®^ (Addinsoft, New York, NY, USA), and log-rank and Wilcoxon tests were performed to determine significant difference in implant survivorship between groups. Data for the KM analysis was censored to the last follow-up date available in the database. 97.0% of the study group had at least 2 years follow-up, and 88.4% had up-to-date follow-up with our protocol listed above. The number of patients remaining at risk were identified on the KM figure.

## Results

KM implant survivorship (Fig. 1) for our study group of patients over 65 at 10 years postoperative was 98.6% and at 16 years was 98.2%. This did not differ significantly from the younger cohort (log-rank *p* value = 0.654 and Wilcoxon *p* value = 0.532), with KM implant survivorship of 99.7 and 98.6% at 10 and 16 years postoperative, respectively. In the study group, implant survivorship for 98.0% for males and 100% for females at 10 years; this did not differ with statistical significance (Log-rank and Wilcoxon *p* value = 0.131).

**Fig. 2:**
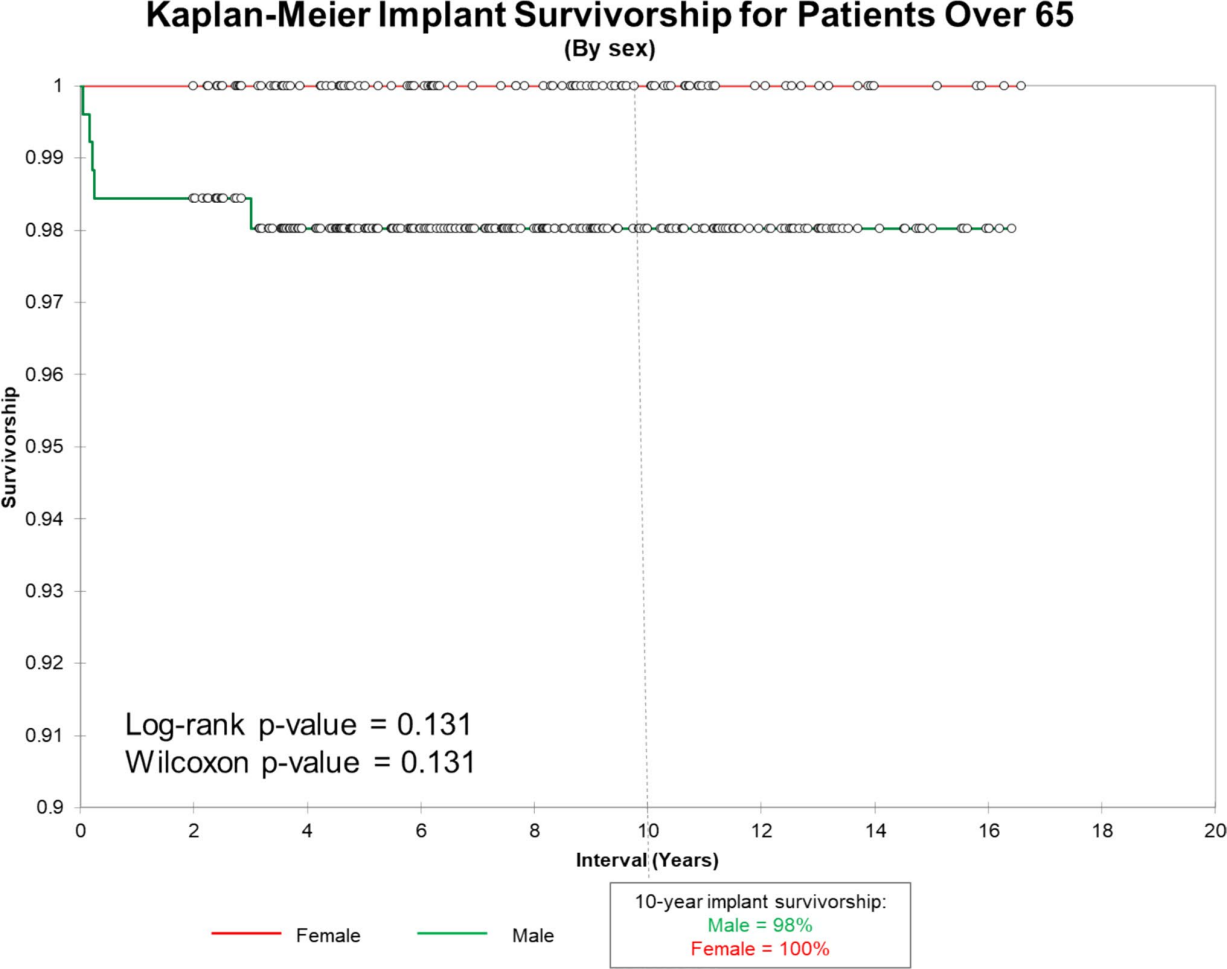
Over-65 Kaplan–Meier Implant Survivorship by Sex

Table 4 lists total failures for both age groups. The only failure modes that varied significantly between the two groups were early femoral fracture (*p* = 0.013) and late infection (*p* = 0.019), which were both higher in the over-65 study group. Overall rate of failure did not differ significantly between the two groups. There were no cases of AWRF or unexplained pain in patients over 65, while both failure modes occurred at a rate of 0.1% in the control group; this did not constitute a statistically significant difference (Fig. 2).

**Table 4:** Failures

Type	< 65 y.o	≥ 65 y.o	*p* value
# Cases	5106	395	**–**
*1) Acetabular Failures*			
Adverse Wear	5 (0.1%)	0 (0.0%)	0.535
Acetabular Loosening (> 2 years)	6 (0.1%)	0 (0.0%)	0.497
Cup Shift	3 (0.1%)	0 (0.0%)	0.631
Failure of Acetabular Ingrowth (< 2 years)	8 (0.2%)	0 (0.0%)	0.430
*2) Femoral Failures*			
Early Femoral Head Collapse (< 6 months)	1 (0.0%)	0 (0.0%)	0.78
Femoral Component Shift	1 (0.0%)	0 (0.0%)	0.78
Femoral Component Loosening (> 1 year)	3 (0.1%)	0 (0.0%)	0.631
Early Femoral Fracture (< 6 months)	14 (0.3%)	4 (1.0%)	0.013*
*3) Other Failures*			
Recurrent Instability	3 (0.1%)	0 (0.0%)	0.631
Early Infection (< 1 year)	0 (0.0%)	0 (0.0%)	1.000
Late Infection (> 1 year)	1 (0.0%)	1 (0.3%)	0.019*
Late Fracture	7 (0.1%)	0 (0.0%)	0.495
Unexplained pain	5 (0.1%)	0 (0.0%)	0.535
Psoas tendonitis	1 (0.0%)	0 (0.0%)	0.78
Other	4 (0.1%)	0 (0.0%)	0.575
Total Failures	62 (1.2%)	5 (1.3%)	0.928

Considering that 11.6% of patients were lost to follow-up or delinquent, we performed a sensitivity analysis assuming that all 46 patients lost to follow-up were implant failures, which yielded a worst-case scenario of 16-year survivorship of 96.9%. In our overall cohort of resurfacings (all ages), a brief follow-up survey of previously non-compliant, lost-to-follow-up patients (n = 561) revealed ongoing implant retention and 99.4% satisfaction, comparable to 96.7% satisfaction in the main up-to-date cohort, suggesting minimal bias from missing data.

Tables 5 and 6 list reoperations and complications, respectively. Reoperations were treated surgically while retaining the original HRA implants. The older study group had significantly more instances of early infection (*p* = 0.04), perhaps suggesting a weaker immune system. The higher rate of associated gluteal tear (*p* = 0.0083) suggests deconditioning. However, the overall rate of reoperation did not differ between groups. Complications were managed with conservative treatment. Rates of infection not resulting in revision surgery did not vary between the two age groups. Overall rate of complications (*p* = 0.28) and reoperation (*p* = 0.28) did not vary significantly between the two age groups.

**Table 5:** Reoperations

Type	< 65 y.o	≥ 65 y.o	*p* value
# Cases	5106	395	**–**
Gluteal Tear	1 (0.02%)	0 (0.0%)	0.78
Early Infection (< 3 months)	6 (0.1%)	0 (0.0%)	0.497
Early infection (< 1 year)	6 (0.1%)	0 (0.0%)	0.497
Late Infection (> 1 year)	1 (0.02%)	0 (0.0%)	0.78
Early fracture (< 6 months)	2 (0.04%)	0 (0.0%)	0.697
Late Fracture (> 6 months)	11 (0.2%)	1 (0.3%)	0.881
Dislocation	1 (0.02%)	0 (0.0%)	0.78
Fascia Failure	4 (0.1%)	0 (0.0%)	0.575
Hematoma	4 (0.1%)	0 (0.0%)	0.575
Peroneal Nerve Palsy	2 (0.04%)	0 (0.0%)	0.697
Psoas Tendonitis	1 (0.02%)	0 (0.0%)	0.78
Other	4 (0.1%)	0 (0.0%)	0.575
Total Reoperations	37 (0.7%)	1 (0.3%)	0.276

**Table 6:** Complications

Type	< 65 y.o	≥ 65 y.o	*p* value
# Cases	5106	395	**–**
Acetabular Component Shift^*^	29 (0.6%)	2 (0.5%)	0.873
Anxiety	3 (0.06%)	0 (0.0%)	0.631
Dislocation	19 (0.4%)	2 (0.5%)	0.674
Early Fracture (< 6 months)	4 (0.08%)	1 (0.3%)	0.267
Late Fracture (> 6 months)	5 (0.1%)	0 (0.0%)	0.535
Early Infection (< 1 year)	1 (0.02%)	0 (0.0%)	0.78
Late Infection (> 1 year)	0 (0.0%)	0 (0.0%)	1.000
Femoral Component Shift	4 (0.08%)	0 (0.0%)	0.575
Hematoma	5 (0.1%)	0 (0.0%)	0.535
Nerve Palsy	5 (0.1%)	3 (0.8%)	0.009*
Spinal Headache	7 (0.1%)	0 (0.0%)	0.495
Urinary Complication	7 (0.1%)	1 (0.3%)	0.562
Cardiovascular Complication	18 (0.4%)	0 (0.0%)	0.238
Nausea/Vomiting	0 (0.0%)	2 (0.5%)	< 0.0001*
Other	16 (0.3%)	2 (0.5%)	0.516
Total Complications	123 (2.4%)	13 (3.3%)	0.276

We categorize periprosthetic fractures in several ways: when they occurred (early vs. late), as well as how they are managed (revised, repaired, treated conservatively). There was a higher early failure rate due to femoral neck fracture (1 vs. 0.3%, *p* = 0.013) in the study group compared to the younger control group, but no significant difference in fracture rate was observed beyond two years postoperative.

Clinical and radiographic results for unrevised cases are presented in Table 3. Postoperative HHS pain scores were no different between the groups but VAS pain scores on regular days (but not worst days) were slightly higher for the study group (mean VAS of 0.4 vs 0.3, *p* = 0.04). The function component of the HHS (85.9 in younger group vs 85.7 in older group, *p* value = 0.7) did not significantly differ between the two groups. However, mean UCLA activity score (control group = 7.5 vs study group = 6.5, *p* < 0.0001) was significantly lower for the older study group.

Approximately 64% of all patients (61.0% of control group and 93.0% of study group patients) complied with ion testing. Table 7 lists ion data for all cases that have not failed. There were 10 AWRF in the control group and none in the study group.

**Table 7:** Metal ion test results

Variables	< 65 y.o		≥ 65 y.o		P values between Groups	
	Unilateral	Bilateral	Unilateral	Bilateral	Unilateral	Bilateral
Co* (µg/L)	1.3 ± 1.0	1.4 ± 1.1	1.4 ± 1.0	1.4 ± 1.1	0.18	1.000
Cr* (µg/L)	1.0 ± 0.9	1.1 ± 0.9	1.0 ± 0.9	1.0 ± 0.9	1.000	0.18
Patients Tested (#, %)	3078/5044 (61.0%)		367/395 (93.0%)		< 0.0001*	
Normal (#,%)	1648 (78.4%)	709 (72.6%)	180 (80.0%)	105 (73.9%)	0.58	0.74
Optimal (#, %)	1950 (92.8%)	891 (91.3%)	217 (96.4%)	133 (93.7%)	0.04*	0.34
Acceptable (#,%)	17 (0.8%)	2 (0.2%)	2 (0.9%)	0 (0.0%)	0.90	0.60
Problematic (#, %)	2 (0.1%)	0 (0.0%)	0 (0.0%)	0 (0.0%)	0.65	1.000
Potentially Toxic (#, %)	0 (0.0%)	0 (0.0%)	0 (0.0%)	0 (0.0%)	1.000	1.000

Average length of incision (*p* = 0.0016), operation time (*p* = 0.017), and estimated blood loss were all lower for the older study group than the remaining control cohort. This may reflect the lower BMI and greater tissue laxity generally seen in older patients, making exposure easier. Mean ASA score (*p* < 0.0001) and mean hospital stay duration (*p* < 0.0001) were both significantly greater for the study group (Table 8).

**Table 8:** Bone management program

	Operative FN	EFF risk (%)	Protocol
High risk	T < -1.5	8	alendronate for 12 months + slow program
Moderate risk	− 1.5 < T < 0	1	alendronate for 6 months
	BMI > 29	5	+ fast program
Low risk	T > 0 + BMI < 30	0.20	fast program and no meds

Table @@ Following this program results in an EFF (early femoral failure rate, all fracture, and head collapse before 1 year) of 0.2%. Femoral neck T-score of the operative femoral neck is used. DEXA scans are done on every patient preoperatively [37, 38]

## Discussion

In this study, we report a 98.6% 10-year and 98.2% 16-year KM implant survivorship for uncemented HRA in 395 patients over age 65 (mean age 68.8 years) performed by a single surgeon. These outcomes were not significantly different from those observed in a younger control cohort (mean age 53) and notably exceed the 10- and 15-year survivorship benchmarks for THA reported by major registries. While some expert-surgeon THA series also outperform registry benchmarks, registry data typically reflect national averages and include a broad range of surgical expertise.

Initially, the senior surgeon of this study reserved HRA for younger patients, especially those with high functional demands. As our experience grew, a greater number of older patients began requesting HRA, particularly those who previously had HRA by this surgeon. Over time, our long-term data demonstrated that HRA in this population yielded desirable outcomes.

Given that older age is often cited as a relative contraindication for HRA in registry and clinical reports, we focused this study on assessing whether age alone should limit candidacy. One factor contributing to early femoral failure (EFF) in older adults, which includes femoral neck fractures and head collapse prior to one year, may be decreased bone density [37, 38]. We previously reported an EFF rate of 2% [31], but through the routine use of DEXA scans and bone management protocols, we have reduced this to 0.2%. In this study, the early fracture rate in the over-65 group was 1.3%. Of these 5 cases, 4 required revision of the femoral component; the remaining case was treated conservatively. These findings suggest that when appropriately managed, low bone density does not prevent successful HRA.

Preservation of the femoral neck in HRA raised theoretical concerns about late fractures in aging patients. However, our data show a low late fracture rate, which compares favorably to published THA data (4.5–7.7% at long-term follow-up) [48, 49]. This may reflect the biomechanical advantage of avoiding a femoral stem, which can contribute to stress shielding proximally and stress concentration distally in THA.

Despite strong clinical outcomes, surgeon hesitation around HRA persists. This is partly due to the technically demanding socket exposure and historical concerns related to MoM implants. Most AWRFs can be linked to poor implant design [25], poor component positioning [50], or corrosion at the THA trunnion rather than the MoM bearing itself. Our study, using only ReCap™–Magnum™ components, reports an AWRF rate of 0.2% overall, with no cases since 2009. We attribute this to adherence to validated component positioning protocols such as the RAIL guideline [35, 36].

While our older patients demonstrated slightly lower UCLA activity scores (6.5 vs. 7.7, p < 0.0001), pain and function scores (HHS and VAS) did not significantly differ. This suggests that while comorbidities may limit overall activity level with age, the function of the resurfaced joint itself remains uncompromised. Some older patients remain highly active and may prefer an implant that enables them to return to sports and high-ROM activities. Our data indicate that HRA in older adults offers implant durability and function equivalent to younger recipients and superior to THA registry benchmarks, provided patients are appropriately selected and surgery is performed by experienced hands.

In addition to implant longevity, other patient-centered outcomes support the use of HRA. Pritchett [1]reported that 86% of patients with both an HRA and a THA preferred the resurfaced side. Other studies show improved gait patterns [2, 3, 5], lower dislocation rates [51], and superior return to demanding activities in HRA patients [6–9, 52]. Importantly, multiple studies have also reported significantly lower all-cause mortality in HRA recipients compared to those undergoing THA [26–30]. This may be related to greater preserved activity levels and exercise tolerance postoperatively. Supporting this, a recent study of 90,000 adults showed that doubling exercise volume or intensity halved all-cause mortality risk [53].

There are notable limitations to this study worth mentioned. First, this is a single-surgeon series by an expert surgeon, so generalizability to lower-volume or less-experienced centers is difficult. Next, the use of a single implant system and strict bone management protocols may not reflect common practice among other surgeons. Lastly, although follow-up rates were high, 11.6% of patients were lost to follow-up; this may introduce data bias.

## Conclusion

To our knowledge, this is the first long-term, large-cohort study demonstrating equivalent implant durability in patients older than 65 to that of younger cohorts. Our implant survivorship of 98.2% at 16 years does not differ significantly from our younger control group and is superior to benchmark registry data for THA. This allows us to offer older patients access the advantages of HRA without the disadvantage of inferior durability that was previously reported. The proven advantages of HRA over THA are a more natural-feeling hip, a more nearly-normal gait, greater stability with high-ROM activity, greater return to impact sports and heavy lifting, and lower all-cause mortality. An unexpected finding from this study was that the total fracture rate of 1.3% was lower than that reported for THA, despite retention of the femoral neck.

These results support a more inclusive approach to resurfacing and challenge longstanding assumptions that age should exclude patients for HRA candidacy. However, due to the steep learning curve of performing resurfacing surgery, we advise that surgeons first gain proficiency with HRA in younger patients before extending indications to older individuals. This study supports that, when performed by experienced surgeons and combined with proactive bone density management, HRA offers a safe, durable, and functionally superior alternative to THA regardless of age.

## Supplementary Information

Below is the link to the electronic supplementary material.

Supplementary file1 (PDF 116 kb)

## Data Availability

No datasets were generated or analysed during the current study.

## Abbreviations

HRA: Hip resurfacing arthroplasty
THA: Total hip arthroplasty
ROM: Range of motion
NJR: British National Joint Registry
MoM: Metal-on-metal
RAIL: Relative acetabular inclination limit
AWRF: Adverse wear-related failure
Co: Cobalt
Cr: Chromium
HHS: Harris hip score
UCLA: University California Los Angeles
VAS: Visual analog score
AIA: Acetabular inclination angle
KM: Kaplan–Meier
EFF: Early femoral failure
