# Supplementary material for: Uncemented hip resurfacing in patients over 65: 16-year outcomes from a large, single-surgeon series
Source: Musculoskelet Surg. 2025 Nov 6;110(3):325–33. doi: 10.1007/s12306-025-00929-2 (PMC13473076; doi:10.1007/s12306-025-00929-2)
Supplement: Supplementary file 1 [file 12306_2025_929_MOESM1_ESM.pdf]

Providence
HEALTH

July 7, 2017

Thomas P. Gross, M.D.
Midlands Orthopedics 1910
Blanding Street Columbia,
SC 29201

Dear Midlands Orthopaedics Research Department:

Manuscripts based on retrospective analyses of OrthoVault data are **exempt** from Institutional Review Board review based on 45 CFR 46, "Collection or Study of Existing Data," taking into account the HIPPA Privacy Rule (45 CFR 160 and 164a) as it pertains to use and disclosure of protected health information.

These manuscripts must be based on retrospective review of existing data that were collected as part and parcel of standard patient care, with no patient identifiers being used in the presentation of data.

Please let us know if we can be of further assistance.

Sincerely,

Charles S. Bryan

Charles S. Bryan, M.D., MACP
Chair, Institutional Review Board
